# Association between Urine Fluoride and Dental Fluorosis as a Toxicity Factor in a Rural Community in the State of San Luis Potosi

**DOI:** 10.1155/2015/647184

**Published:** 2015-02-19

**Authors:** Lizet Jarquín-Yañez, José de Jesús Mejía-Saavedra, Nelly Molina-Frechero, Enrique Gaona, Diana Olivia Rocha-Amador, Olga Dania López-Guzmán, Ronell Bologna-Molina

**Affiliations:** ^1^Center of Applied Research in Environment and Health, CIACYT, School of Medicine, Autonomous University of San Luis Potosi, 78100 San Luis Potosi, SLP, Mexico; ^2^Division of Biological and Health Sciences, Metropolitan Autonomous University, 04960 Mexico City, MEX, Mexico; ^3^Division of Natural and Exact Sciences, University of Guanajuato, 36050 Guanajuato, GTO, Mexico; ^4^School of Chemical Sciences, Durango Unit, Juarez University of the State of Durango, 3400 Durango, DGO, Mexico; ^5^Department of Research, School of Dentistry, Juarez University of the State of Durango, 34000 Durango, DGO, Mexico

## Abstract

*Objective*. The aim of this study is to investigate urine fluoride concentration as a toxicity factor in a rural community in the state of San Luis Potosi, Mexico. *Materials and Methods*. A sample of 111 children exposed to high concentrations of fluoride in drinking water (4.13 mg/L) was evaluated. Fluoride exposure was determined by measuring urine fluoride concentration using the potentiometric method with an ion selective electrode. The diagnosis of dental fluorosis was performed by clinical examination, and the severity of damage was determined using Dean's index and the Thylstrup-Fejerskov (TF) index. *Results*. The range of exposure in the study population, evaluated through the fluoride content in urine, was 1.1 to 5.9 mg/L, with a mean of 3.14 ± 1.09 mg/L. Dental fluorosis was present in all subjects, of which 95% had severe cases. Higher urine fluoride levels and greater degrees of severity occurred in older children. *Conclusions*. The results show that dental fluorosis was determined by the presence of fluoride exposure finding a high positive correlation between the severity of fluorosis and urine fluoride concentration and the years of exposure suggested a cumulative effect.

## 1. Introduction

There are contaminants in the environment in constant interaction with us that can affect our health through exposure to them. Drinking water can transmit numerous diseases caused by different pollutants; two of the most common chemicals in water that are capable of causing health problems are fluoride and arsenic. Fluoride (F^−^) is a toxic agent that causes adverse health effects, such as dental and skeletal fluorosis, reproductive and neurological effects, and endocrine disorders [1, 2].

In 1936, it was shown that the increase of fluoride content in water causes dental fluorosis, which is an alteration of tooth enamel that can be observed as spots ranging from whitish to dark brown color and that, in severe cases, leads to the loss of tooth enamel [3]. Research suggests that fluoride affects enamel formation by making it porous. Skeletal fluorosis is a condition associated with the accumulation of fluoride in bone, resulting in brittle bones that are susceptible to tensile forces [4].

Furthermore, studies conducted in recent years suggest that fluoride is a neurotoxic agent, as research conducted in populations exposed to F^−^ (with water concentrations higher than 3 mg/L) supports the hypothesis that F^−^ decreases the intelligence quotient (IQ) of children [5, 6].

Groundwater fluoride contamination is present in 17 states in central and southwestern Mexico; in San Luis Potosi, fluoride is found naturally in high concentrations in water extracted from deep underground wells that are used for human consumption [7–9]. In a study over a decade ago there were already reports in the literature of the existence of high concentrations of fluoride in water in municipal wells in the state of San Luis Potosi with fluoride levels detected of 4.0 mg/L [10].

Dental fluorosis is a public health problem that affects the child population during the period of hard tissue formation. Its prevalence in San Luis Potosi is higher than normal; a rate of 69% was found in populations with water fluoride levels lower than 0.7 mg/L and a rate of 98% in populations with fluoride levels of 2 mg/L [10]. However, water cannot be considered the only source of fluoride exposure. Fluoride-iodized salt, certain beverages such as soda and fruit juices, and toothpaste are other factors that contribute to exposure [11].

As the main source of fluoride is drinking water, the community of La Reforma, Salinas de Hidalgo, San Luis Potosi, is at high risk of developing diseases because it exceeds the permissible limit of fluoride in drinking water of 1.5 mg/L established by the NOM-127-SSA1-1994 [12]. Having waters with high fluoride concentration of 4.13 mg/L, as reported by COEPRIS [13] (State Commission for Protection against Health Risks), this community has one of the highest fluoride concentrations in the state. In addition, the high degree of marginalization in the community results in poor access to high-quality bottled water [14]. Moreover, the dry climate in the region [15] requires heightened water consumption.

High concentrations of fluoride in the community water prompted this study, the aim of which is to evaluate urine fluoride concentration as a toxicity factor of dental fluorosis in a rural community in the state of San Luis Potosi, Mexico.

## 2. Materials and Methods

### 2.1. Study Population

The study was conducted in the community of La Reforma, Salinas de Hidalgo, in the state of San Luis Potosi, Mexico; this is an area with people of low-socioeconomic status. The community was selected due to having fluoride levels in its drinking water of 4.13 mg/L (COEPRIS, 2012) [13], which is higher than both the Mexican standard (1.5 mg/L) [12] and the level recommended by the World Health Organization (1 mg/L) [2]. The total population from 6 to 12 years were selected (one hundred and eighty school children).

The study was free, anonymous, and voluntary, and it complied with all requirements of the Bioethics Committee of the School of Medicine of the Autonomous University of San Luis Potosi.

A questionnaire was applied to the children's mothers to collect information regarding exposure to fluorides and collect information about the origin of the drinking water and if this water was used for food preparation.

The inclusion criteria were as follows: having obtained the informed consent of parents or guardians, being born and raised in the study area, having permanent dentition, and providing a sufficient amount of urine to be analyzed. The exclusion criterion was the presence of kidney diseases. Because not all children met the requirements of the study, only 111 children who met the criteria were ultimately incorporated into the study.

The subjects were male 52.3% and female 47.7%, mean age 9.14 ± 1.98 years. The children were divided into three age groups: 6 and 7 years old (35), male 51.4 and female 48.6%; 8–10 years old (37), male 45.9 and female 54.1; and 11-12 years old (39), male 59 and female 41%.

To confirm fluoride levels in the drinking water in the community, drinking water samples provided by the participants were analyzed; in these samples, a mean fluoride level of 4.54 ± 0.46 mg/L was recorded.

### 2.2. Urine Samples

The collection of urine samples from each of the participants was performed in plastic containers previously washed with 10% HNO_3_, collecting the first morning urine. To quantify the fluoride ions, the potentiometric method with an ion selective electrode according to method 3808 of the US National Institute for Occupational Safety and Health [16] (NIOSH) was used. A calibration curve was constructed from 0.1 to 10.0 mg/L. Samples were mixed with total ion strength adjustment buffer (TISAB) in a 1 : 1 ratio for quantification. Finally, the fluoride concentration of the sample was determined by interpolation of the potential in the calibration curve. As a quality control, SRM 2671^a^ “Fluoride in Freeze-Dried Urine” (NIST) reference material was used with a recovery of 97 ± 6%. The correction for urine dilution was performed based on specific gravity [17].

### 2.3. Clinical Evaluation

Clinical dental examination was performed according to the requirements outlined by WHO for national oral health surveys, 1997 [18], taking 10 minutes as reference time duration for the basic examination of a child. The test area was prepared with the required hygiene and safety measures, using previously sterilized instruments and having easy access to sterilization procedures and using a plane mirror and a periodontal probe.

The diagnosis of the degree of dental fluorosis was performed by applying Dean's index (DI) [18, 19] and the Thylstrup-Fejerskov index (TF) [20], which is more sensitive for individual classifications of dental organs in ten categories. A score of zero indicates healthy enamel; scores of one to four indicate spots on the enamel surface, which increase as the score increases. Enamel destruction is observed in scores five to nine, where score five represents mottled enamel with holes smaller than 2 mm in diameter, which are fused in score six to form bands less than 2 mm deep. Scores of seven, eight, and nine represent the destruction of 25%, 50%, and 100% of the enamel surface.

Interexaminer and intraexaminer calibration were performed by two examiners (>0.89 Kappa). The community index of dental fluorosis was obtained by multiplying the number of children in each Dean's score by their weightings, adding the results and dividing by the number of children examined.

### 2.4. Statistical Analysis

Data collected from oral clinical assessment and urine fluoride levels were transferred into a Microsoft EXCEL database, which was then analyzed in SPSS version 21. Variables were assessed using univariate analysis to obtain percentages and distributions. Means, standard deviations, and confidence intervals were analyzed. Frequency distribution by age group and analysis of variance between the TF dental fluorosis index and the age groups were performed. To establish the correlation between urine fluoride concentration and severity of dental fluorosis, the Spearman correlation coefficient was used.

## 3. Results

The minimum level of fluoride in urine was 1.1 mg/L, which was present in only two children in the group with ages of 6 and 7 years. The maximum exposure level was 5.9 mg/L, which was also present in two children but with ages of 9 and 11 years. The mean urine fluoride concentration of the population was 3.14 ± 1.09 mg/L.

Table 1 shows the severity of dental fluorosis in the tree age groups with confidence intervals.

The urine fluoride concentration was classified into four levels: 1 to 2 mg/L, 2.01 to 3 mg/L, 3.01 to 4 mg/L, and >4 mg/L.

**Figure 1:**
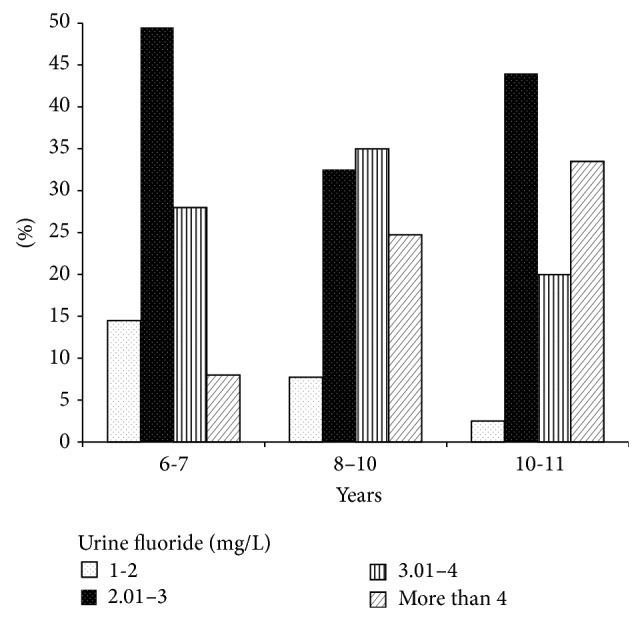
Urine fluoride concentration in children in the study area by age group as percentage.

Figure 1 shows the distribution of the urine fluoride levels of the children, noting that at the ages of 6 to 7 years, fluoride excretion ranged between 2.01 and 3 mg/L (48.6%), with a low prevalence of high fluoride concentrations (5.7%). In the age group of 8–12 years, the prevalence of high fluoride concentrations increased, and the prevalence of low fluoride concentrations (1 and 2 mg/L) decreased.

The information collected through questionnaire reported that 100% of population used tap water for cooking and 83.8% for drinking.

All children examined showed dental fluorosis; only 5% of the children had a moderate score, while the rest (95%) had a severe score based on Dean's index. According to the TF, the score TF4 occurred in 4.5% of the population, TF5 in 25.2%, TF6 in 16.2%, TF7 in 28.8%, TF8 in 16.2%, and TF9 in 9%.

Children with TF4 and TF5 constituted 30% of the study population, TF6 and TF7 45%, and TF8 and TF9 25%.

**Figure 2:**
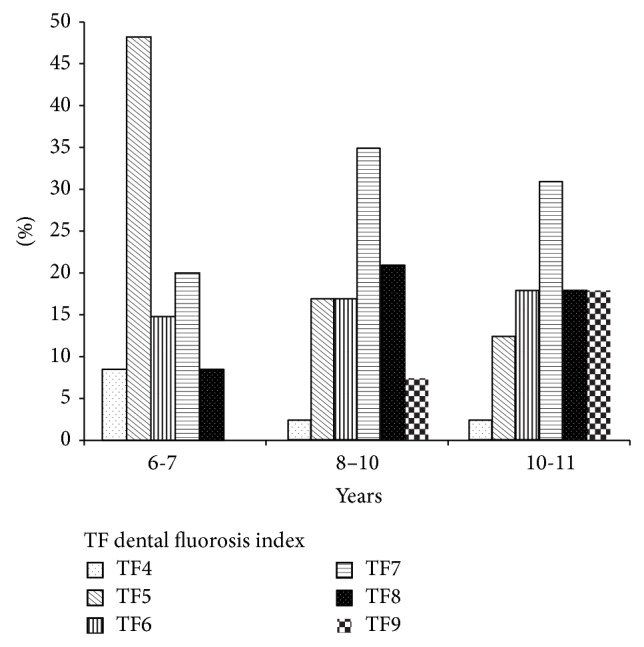
Distribution of dental fluorosis according to the TF index by age group as percentage.

At the ages of 6 and 7 years, TF4 and TF5 predominated at 57.14%, with a predominance of TF5 in 48.6%; TF6 and TF7 predominated in 76% of children aged 8 to 12 years, while the levels of greater severity, such as TF8 and TF9, were observed in 50% of the children at the ages of 11 and 12 years. This result is somewhat related to the exposure level, as children with higher urine fluoride levels were 8 and 12 years old. There were significant differences between age groups and the corresponding severity of dental fluorosis *P* < 0.000 (Figure 2).

**Figure 3:**
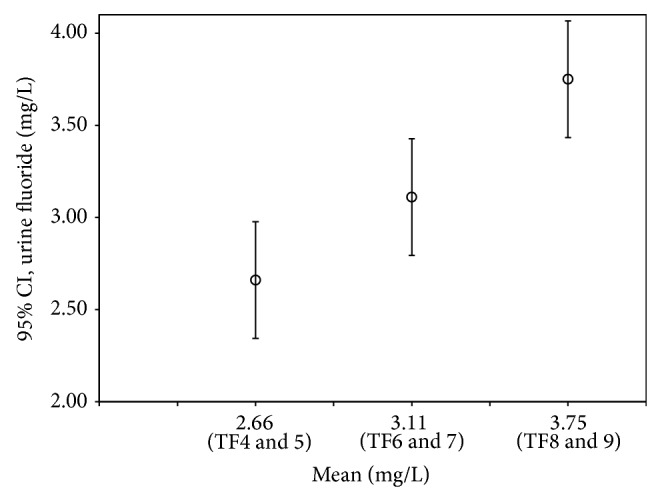
Urine fluoride levels and their relation to dental fluorosis scores.

In relation to the presence of fluoride in urine and the severity of dental fluorosis, Figure 3 shows urine fluoride levels with mean values of 2.66 ± 0.89 (95% CI 2.35–2.98) in the 33 children with TF4 and TF5, 3.11 ± 1.06 (95% CI 2.81–3.41) in the 50 children with TF6 and TF7, and 3.75 ± 1.10 (95% CI 3.32–4.18) in the 28 children with TF8 and TF9, with a high positive correlation between the severity of fluorosis and the urine fluoride concentration *r* = 0.99 and ^**^ *P* < 0.01.

## 4. Discussion

The concentration of fluoride in drinking water in this community was found to be higher than both the Mexican standard (1.5 mg/L) and the level suggested by the WHO (1 mg/L), with a mean level of 4.54 mg/L ± 0.46, which is even higher than the level reported in the same area in 2012 by COEPRIS [13], most likely due to the depletion of the aquifer.

Urine fluoride concentration ranged from 1.1 to 5.9 mg/L, with the lowest levels of exposure in children aged six to seven years, a period in which children are growing and with increased retention of fluoride in hard tissues, such as teeth and bones. Most fluoride exposure occurred in the older group.

The study conducted by Grimaldo et al. [8] in the city of San Luis Potosi determined risk factors associated with human fluoride exposure through drinking water; preparing food with tap water was one of them. This risk factor was also present in this study; as the location was a rural area of high marginalization, 83.8% of the study population used tap water for drinking, and 100% used it for cooking.

The children with higher urine fluoride levels (more than 4 mg/L) were aged 8 and 12 years. This result is possibly due to the longer exposure time and the cumulative effect of fluoride stored in bones and teeth, particularly in growing organisms due to the greater fluoride retention in children's bones, which is caused by high blood flow and a large area of hydroxyapatite crystals unlike mature bone.

Due to the fluoride water concentration in the area, dental fluorosis in this study was severe in 95% of the study population, in agreement with the results of the study conducted by Grimaldo et al. [9] in 1997 in the city of San Luis Potosi. These authors reported a prevalence of dental fluorosis in 96% of children aged 11 to 13 years in an area with a mean fluoride concentration of 3.29 mg/L. In contrast, in the current study, the prevalence was 100% at a higher fluoride concentration in drinking water and in younger children. Our study determined not only the prevalence of dental fluorosis but also the exposure level and the assessment of the degree of severity of dental fluorosis according to the TF index.

The degrees of greater severity of dental fluorosis TF8 and TF9 occurred in a higher percentage in children aged 11 and 12 years, most likely due to longer exposure to fluoride and the presence of a greater number of permanent teeth.

Fluorosis is irreversible, as the teeth remain fragile and susceptible to rupture, and prosthetic dental treatments that stop tooth destruction are expensive; thus, patients and their families often cannot afford them, leading to the loss of teeth and consequent effects on the individual's quality of life.

The present work showed the limitations of a cross-sectional study. For accuracy, the urine collection should be done over a period of 24 hours.

Our results show the close relationship between fluoride exposure caused by high intake of fluoride through drinking water and the severity of dental fluorosis, which affects the quality of life of the population studied. There is a need for further studies in this community to identify other factors that are exacerbating exposure.

## Figures and Tables

**Table 1:** Sample in number and percentage for groups of age and mean of dental fluorosis using TF index.

	Male	Female
Ages 6-7		
Number	18	17
Percentage	51.4	48.6
TF (95% CI)	5.5 (4.95–6.0)	5.94 (5.33–6.56)
Ages 8–10		
Number	17	20
Percentage	45.95	54.1
TF (95% CI)	6.59 (5.9–7.3)	7.0 (6.5–7.5)
Ages 11-12		
Number	23	16
Percentage	58.97	41.03
TF (95% CI)	7.04 (6.48–7.6)	7.0 (6.2–7.8)

TF: Thylstrup-Fejerskov index.

CI: confidence interval.
